# Fermentation Technology in Aquafeeds: A Review

**DOI:** 10.3390/microorganisms14020416

**Published:** 2026-02-10

**Authors:** Haoxuan Sun, Linwei Nie, Miaoqin Huang, Weiguang Zou, Yu Hu, Xuan Luo, Weiwei You, Caihuan Ke

**Affiliations:** 1 State Key Laboratory of Mariculture Breeding, College of Ocean and Earth Sciences, Xiamen University, Xiamen 361102, China; haoxuansun0917@stu.xmu.edu.cn (H.S.);; 2 Fujian Key Laboratory of Genetics and Breeding of Marine Organisms, Xiamen University, Xiamen 361102, China

**Keywords:** aquafeeds, fish, aquaculture, fermented feed, gut health

## Abstract

Fermented feed, as a novel type of functional feed, enhances the nutritional value and palatability of feed through microbial fermentation. It effectively mitigates issues such as impaired digestive function, compromised immune response in aquaculture animals, and the over-reliance on fishmeal-based diets. This paper provides a narrative review of the fermented feed, its benefits, its common types, and the factors influencing its efficacy. Furthermore, it summarizes its beneficial effects in aquaculture animals, including growth promotion, immune enhancement, optimization of intestinal microbiota, and improvement in product quality. Despite its considerable potential to reduce costs and improve efficiency, fermented feed faces challenges such as limited industrial-scale production and little research on crustaceans. Future research should focus on database development, intelligent manufacturing processes, and comprehensive economic evaluations to facilitate its broader application in aquaculture.

## 1. Introduction

Aquaculture animals represent a significant source of dietary protein for humans, and aquaculture constitutes a crucial economic sector. Compared to terrestrial livestock such as pigs and chickens, aquaculture animals exhibit distinct characteristics, including limited digestive and absorptive capacities, reduced disease resistance and stress tolerance [1], challenges in disease detection and diagnosis [2], and elevated protein requirements [3]. Compared to terrestrial livestock, aquaculture animals present distinct physiological and husbandry challenges. Their digestive and immune systems have evolved in aquatic environments, which differ fundamentally from the conditions of intensive aquaculture [4]. In high-density or super-intensive farming systems, these inherent traits—such as specific dietary requirements, a reliance on waterborne ion exchange, and often subtle disease symptoms—can heighten susceptibility to nutritional imbalances and pathogens. Furthermore, the aquatic medium itself complicates the direct observation and timely diagnosis of health issues [5]. Accordingly, disease prevention and enhancement of immune function are critical for sustainable aquaculture. Additionally, aquaculture animals exhibit high protein deposition and correspondingly high demands for exogenous protein intake [3]. Compared to terrestrial livestock, their feeds necessitate a greater protein content; for example, crude protein levels in broiler and finishing pig feeds are approximately 21% [6] and 15% [7], respectively, whereas feeds for grass carp and abalone contain around 32% [8] and 36% crude protein [9]. Aquaculture animals also exhibit limited utilization of fats and carbohydrates [10], often relying on protein as an energy source. Consequently, protein feed, representing the largest cost component, is vital in aquaculture nutrition, making the development and utilization of novel protein sources a key focus in feed research.

Addressing the aforementioned challenges faced by aquaculture animals, the research, development, and application of fermented feed offer an effective solution. Fermented feed is produced by the microbial fermentation of conventional feedstuffs, resulting in enhanced feed resources. It confers multiple advantages: (1) improving feed quality and palatability, thereby increasing feed intake and promoting feed efficiency in aquaculture animals [11]; (2) converting macromolecular compounds such as proteins and carbohydrates into smaller molecules like acid-soluble proteins and reducing sugars, which facilitates nutrient digestion and absorption, supports intestinal health, and enhances immunity and disease resistance [12]; and (3) improving the physicochemical properties of the feed and degrading anti-nutritional factors. Consequently, fermented feed represents a novel feed resource; for instance, fermented protein feeds have demonstrated potential as partial or complete substitutes for fishmeal [13].

Consequently, research on fermented feed has emerged as a prominent focus within the field of feed science, offering effective solutions to challenges in the aquaculture industry. This paper reviews the characteristics of fermented feed, factors influencing fermentation, its application effects and existing limitations, as well as current research trends, with the aim of providing a theoretical foundation to guide relevant production and practical applications.

## 2. Characteristics of Fermented Feed

Biological fermentation technology enhances fermented feed by not only improving its nutritional value [14] but also increasing the abundance of beneficial bacteria through the activity of fermentation strains [15]. Fermented feed offers multiple benefits, including improving feed palatability, promoting growth and development, enhancing digestibility [16], supporting intestinal health [17], degrading mycotoxins [18], improving product quality [7], protecting the environment [19], breaking down anti-nutritional factors [20], reducing animal mortality [21], and extending feed shelf life [22]. The following sections discuss the benefits, common types of fermented feed.

### 2.1. Benefits of Fermented Feed

Microbial activity is central to the feed fermentation process [23]. Fermented feed contains beneficial active microorganisms that not only inhibit the growth of pathogenic bacteria within the animal digestive tract, thereby maintaining intestinal health [24], but also degrade anti-nutritional factors present in certain feed raw materials. These factors, such as gossypol, mannan, and phytic acid, can impair nutrient absorption. Through microbial fermentation, these inhibitors are broken down, leading to improved nutritional value of the feed [25].

**Figure 1:**
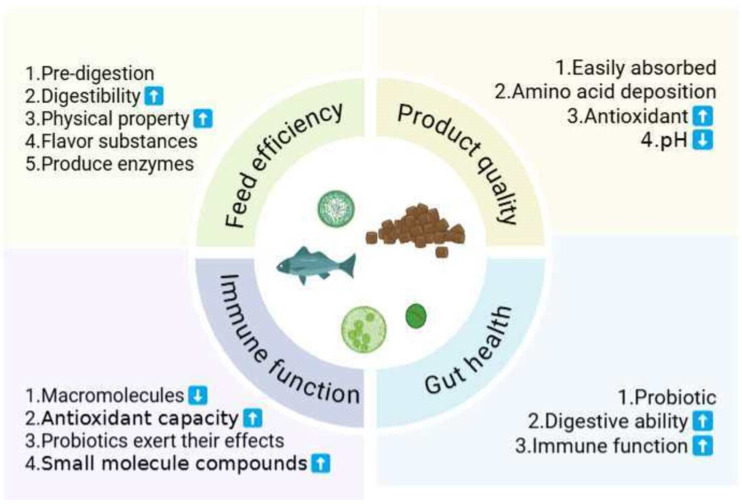
The benefits of fermented feed on aquaculture animals.

Fermentation of protein feed serves as a pre-digestion process. During fermentation, enzymes secreted by microorganisms break down complex proteins into shorter peptides and simpler amino acids [26]. This pre-digestion facilitates protein absorption by animals and enhances feed utilization efficiency. Additionally, fermentation improves feed pH and flavor, increasing palatability. Organic acids produced during fermentation further promote digestive tract health and enhance feed digestibility [27]. Moreover, microorganisms synthesize vitamins—such as B-group vitamins—and other bioactive compounds during fermentation, which contribute to improved immunity and growth performance in animals [27,28]. The benefits of fermented feed is shown in Figure 1.

### 2.2. Common Categories of Fermented Feed

Fermented feeds are primarily categorized into fermented protein feeds, fermented energy feeds and fermented roughage. Fermented roughage, mainly used for ruminants such as cattle and sheep, is not addressed in this paper. Fermented protein feeds are further subdivided into fermented plant protein feeds, fermented animal protein feeds, and microbial protein feeds, with fermented plant protein feeds comprising the majority of total usage [29]. Common examples of fermented plant protein feeds include soybean meal, cottonseed meal, rapeseed meal, and peanut meal [30]. Fermented animal protein feeds consist of meat and bone meal, blood meal, feather meal, and insect protein. Compared to plant protein feeds, animal protein feeds have higher protein content and a more balanced amino acid profile, although they are generally more expensive [31].

**Figure 2:**
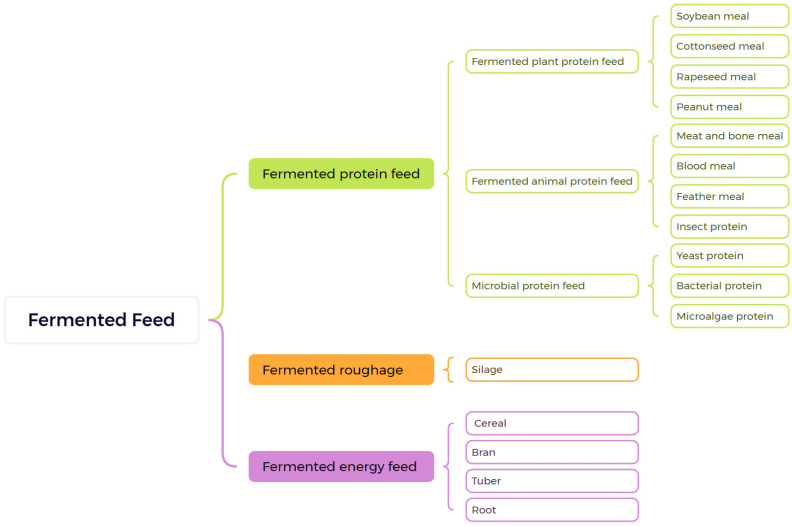
The common categories of fermented feed.

Microbial protein feeds, also referred to as single-cell proteins, encompass yeast protein, bacterial protein, and microalgae protein. These feeds have diverse sources and can be produced using industrial waste, offering efficient production methods and multifunctional benefits [3,32]. Fermented energy feeds include fermented cereals, bran, tubers, roots, and other energy-rich materials. Common fermented energy feeds are corn, wheat bran, mushroom bran, and cassava residue. The common categories of fermented feed are shown in Figure 2.

## 3. Factors Affecting the Quality of Fermented Feed

The quality of fermented feed is influenced by various factors, with the bacterial strain being the most critical, as fermentation fundamentally depends on microbial activity [33]. Other important factors affecting feed fermentation using bacterial strains, in order of significance, include temperature, fermentation duration, and moisture content [34]. The following sections discuss these four key factors in detail.

### 3.1. Strain

The selection of microbial strains is a decisive factor influencing the fermentation process, often regarded as the “soul” of fermentation. It directly determines the direction, efficiency, and final composition of the fermentation products. The types of microorganisms used—particularly bacteria and fungi such as yeasts and molds—significantly affect the fermentation outcomes. These microorganisms influence the functional, nutritional, and sensory properties of the final products [35]. Commonly employed fermentation strains include yeasts, lactic acid bacteria, and *Bacillus* spp. [36]. The specific microorganisms involved dictate the fermentation type and metabolic pathways, thereby affecting the physical characteristics of the product. Notably, modern fermented feeds often utilize compound microbial consortia to leverage the synergistic effects arising from strain combinations [37].

The dominant microorganisms during fermentation determine its trajectory. For instance, lactic acid bacteria primarily drive lactic acid fermentation, rapidly producing acid to lower pH, inhibit spoilage bacteria, and acidify the feed, aiming to preserve nutrients and prevent spoilage [38]. Yeasts facilitate alcoholic fermentation, generating ethanol, CO_2_, and flavor compounds that enhance feed palatability and probiotic properties, although their preservation effect is generally less effective than lactic acid fermentation [32]. Bacillus species produce potent enzymes such as proteases, amylases, and cellulases that extensively break down macromolecular nutrients, significantly improving digestibility and potentially producing antimicrobial peptides [39]. Molds secrete cellulases and hemicellulases, effectively degrading fibrous materials and enhancing feed utilization [40].

Different bacterial strains can influence the physical properties, preservation, and palatability of feed. For example, certain specialized strains, such as specific lactic acid bacteria and yeasts, possess the ability to degrade mycotoxins, thereby reducing toxin-related harm [41]. Fermentation with lactic acid bacteria can significantly prolong feed preservation and prevent secondary fermentation or mold growth [42]. Moreover, various fermentation strains can improve feed texture; microorganisms that degrade fiber and pectin often soften the feed and facilitate mixing [43]. Microbial fermentation also enhances feed flavor through different mechanisms. For instance, lactic acid and acetic acid produce a sour aroma, while yeasts generate ester-like and mellow aromas, collectively improving the sensory appeal and increasing animal feed intake [44]. Beyond flavor enhancement, fermentation can reduce unpleasant odors by inhibiting spoilage bacteria responsible for producing malodorous compounds such as ammonia, cadaverine, and putrescine [45].

### 3.2. Temperature

In addition to the internal factor of bacterial strains, temperature is a critical external environmental factor influencing fermentation, as it regulates both the activity and community structure of microorganisms [46]. Each microorganism exhibits a specific optimal temperature range for growth and metabolism [47]. At temperatures that are too low, microbial metabolic activity slows significantly or ceases entirely, leading to a delayed fermentation process and an extended fermentation cycle. This can prevent the achievement of target acidity or active ingredient levels, resulting in incomplete or unsuccessful fermentation [46]. Moreover, harmful microorganisms may also persist or grow slowly under low-temperature conditions [48]. Conversely, excessively high temperatures exceeding microorganisms’ upper tolerance limits can kill or severely inhibit beneficial microbes [49]. High temperatures may also favor the proliferation of heat-resistant harmful bacteria and accelerate enzyme inactivation, further compromising fermentation quality [50].

Regarding the regulation of microbial community structure, the growth advantages and competitiveness of different microorganisms vary with temperature. For instance, moderate temperatures (e.g., 30 °C to 40 °C) typically provide optimal conditions for lactic acid bacteria and yeasts, promoting rapid acid production, pH reduction, and suppression of undesirable bacteria [51]. Consequently, fermentation temperature should be selected based on the desired microbial targets. Appropriate temperature control is essential to encourage the growth of specific dominant microbial communities, while improper control can result in the proliferation of non-target microorganisms, ultimately compromising the quality and functionality of the final product [52].

### 3.3. Fermentation Duration

Fermentation time is a crucial factor in regulating the effectiveness of fermented feed by controlling the fermentation process. An appropriate duration allows microorganisms to proliferate sufficiently and produce beneficial metabolites, such as organic acids, amino acids, and enzymes, thereby enhancing the digestibility and nutritional value of the feed [53]. If the fermentation time is too short, microbial activity may be inadequate to fully convert the nutrients in the raw materials [54]. Conversely, excessively long fermentation can lead to over-fermentation, causing nutrient loss or the accumulation of harmful substances [55].

Fermentation time also influences the extent of decomposition of complex organic matter in raw materials. During the initial fermentation stage, microorganisms break down macromolecules such as carbohydrates and proteins into simpler nutrients, thereby enhancing the bioavailability of the feed [56]. However, excessive fermentation time can lead to over-decomposition of nutrients like proteins, causing the loss of beneficial compounds [57].

### 3.4. Moisture Content

Moisture content is a critical factor in the fermentation of feed, significantly influencing both the fermentation process and nutrient utilization. Adequate moisture provides a conducive environment for microbial growth. Beneficial microorganisms, such as lactic acid bacteria and yeasts, can rapidly proliferate, produce acids, and suppress undesirable bacteria under optimal moisture conditions. Additionally, water facilitates the dissolution and transport of substrates, including carbon sources, essential for microbial metabolism [15]. Appropriate moisture levels ensure efficient nutrient flow and timely diffusion of metabolic products like lactic acid [58]. In fermentations dominated by lactic acid bacteria, optimal moisture compacts the raw materials, expels air, and creates an anaerobic environment favorable for lactic acid bacteria growth. Conversely, excessive moisture can lead to feed spoilage, nutrient loss, and undesirable odors and palatability. Insufficient moisture restricts microbial activity, resulting in incomplete fermentation, a hard texture, increased dust, and reduced feed intake by animals [59].

### 3.5. Fermentation Type

The availability of oxygen during fermentation is one of the fundamental factors determining microbial community succession, metabolic pathways, and the characteristics of the final product—a factor no less critical than strain selection.

Aerobic Fermentation: This process proceeds under conditions of sufficient oxygen supply. It is generally conducive to the growth of obligate aerobic or facultative anaerobic microorganisms, such as many Bacillus spp. and molds (e.g., Aspergillus oryzae) [60]. Under aerobic conditions, microorganisms carry out efficient aerobic respiration, enabling the rapid and thorough breakdown of substrates (such as proteins and cellulose). This process yields a substantial amount of enzymes, microbial protein, and carbon dioxide, while also releasing considerable heat. The primary aim is to maximize both bioconversion and substrate degradation [61].

Anaerobic Fermentation: This process occurs under strictly oxygen-limited conditions. It represents the preferred environment for strict anaerobes or facultative anaerobes, such as lactic acid bacteria and clostridia. In lactic acid bacteria-dominated anaerobic fermentation, organic acids like lactic acid and acetic acid are rapidly produced via homofermentative or heterofermentative pathways, leading to a swift decrease in pH. The primary objectives are to preserve feed, inhibit spoilage microorganisms, and acidify the product [62]. Alcoholic fermentation by yeast also predominantly takes place under anaerobic conditions [63].

Safety Considerations: Notably, improper anaerobic conditions (e.g., failure in pH control) may also lead to the predominance of *Clostridium* species, which can produce butyric acid, biogenic amines, or even toxins, thereby posing safety risks. Table 1 summarizes specific conditions related to fermentation strains, monitoring microbial titer, nutrient profile changes, fermentation type, temperature, fermentation duration, and moisture reported in recent studies on fermented feed.

**Table 1:** Fermentation parameters of selected feed materials.

Fermentation Substrate	Strains	Temperature	Fermentation Duration	Moisture Content	Fermentation Type	Nutrient Profile Changes	Monitoring Microbial Titer	Experimental Animals	References
Silkworm pupae	Lactic acid bacteria	26 ± 2 °C	3 days	60.2%	Anaerobic	CP from 25.5 to 22.9	Not reported	30% each of catla (*Catla catla*), mrigal (*C.mrigala*) and rohu (*L. rohita*) with 10% silver carps (*Hypophthalmychthys molitrix*)	[64]
Black soldier fly larvae	*Lactobacillus agilis*	25 °C	3 days	72%	Anaerobic	Initial CP 45%	Not reported	Asian swamp eel (*monopterus albus*)	[65]
Soybean meal	*Monascus purpureus*	30 °C	10 days	20%	Aerobiotic	CP from 46.0% to 51.6%	Not reported	Pacific white shrimp (*Litopenaeus vannamei*)	[66]
Poultry by-product meal	*Saccharomyces* *cerevisiae*	28–30 °C	4 days	60–65%	Facultative anaerobic	CP from 60.2% to 65%	2.27 × 10^10^ CFU/g	Nile tilapia (*Oreochromis niloticus*)	[67]
The mixture of abalone waste and Sargassum spp (9:1)	*Saccharomyces cerevisiae* and *Lactobacillus casei*	30 °C	4 days	70%	Mixed	CP from 27.5% to 27.5%	Not reported	Marron (*Cherax cainii*)	[17]
Corn cob	*Lactiplantibacillus plantarum*	30 °C	3 days	6%	Facultative anaerobic	Final CP 12%	1 ×10^7^–10^8^ CFU/g	Nile tilapia (*Oreochromis niloticus*)	[68]
Wheat, soybean Meal, corn gluten meal, chicken meal	*C.somerae, S.cerevisiae, L.rhamnosus* and *B. subtilis*	37 °C	3 days	22%	Mixed	CP from 44.9% to 45.5%	1 ×10^10^ CFU/g, 5 × 10^9^ CFU/g, 5 × 10^10^ CFU/g and 5 × 10^9^ CFU/g	Common carp (*Cyprinus carpio*)	[69]
Sesame Seed meal	*Lactobacillus plantarium*	30 °C	2 days	33%	Facultative anaerobic	CP from 23% to 24.4%	Not reported	Til-aqua natural male tilapia	[70]
Mulberry leaf meal	*Stenotrophomonas maltophilia*	37 °C	10 days	80%	Aerobiotic	CP from 26.2% to 19.2%	Not reported	Catfish (*Heteropneustes fossilis*)	[71]
Soybean meal	*Bacillus pumilus* and *Pseudozyma aphidis*	30 °C	2 days	50%	Aerobiotic	CP from 50.6% to 58.5%	1 × 10^5^ CFU/g	Japanese seabass (*Lateolabrax japonicus*)	[72]
Soybean meal	*B.* *pumilus*	37 °C	1.5 days	54%	Aerobiotic	CP from 46.8% to 55.2%	Not reported	Coho salmon (*Oncorhynchus kisutch*)	[73]
Pomelo Peel and Soybean Meal	*B. pumilus*, *Saccharomyces cerevisiae, Lactococcus lactis*	30 °C	2 days	Not reported	Mixed	Final CP 47.76%	6 × 10^5^ CFU/g, 7 × 10^5^ CFU/g, and 2.25 × 10^4^ CFU/g.	Large Yellow Croaker (*Larimichthys crocea*)	[74]
Soybean meal	*Lactobacillus plantarum*	37 °C	3 days	15%	Anaerobic	Initial CP 45%	7 × 10^8^ CFU/g	Abalone (*Haliotis discus hannai*)	[75]
Soybean meal	*B.* *subtilis*	40 °C	3 days	50%	Aerobic	CP from 43.4% to 50.7%	1 × 10^7^ CFU/g	Nile tilapia (*Oreochromis niloticus*) fingerlings	[13]
Olive Mill and Winery By-Products	*Aspergillus ibericus*	25 °C	7 days	75%	Aerobic	Initial CP 48%	Not reported	Fish	[22]
Soybean meal	*E.* *faecium*	37 °C	2 days	55%	Facultative anaerobic	CP from 53.0% to 54.5%	1 × 10^8^ CFU/g	Turbot (*Scophthalmus maximus* L.)	[76]

Note: CP stands for crude protein; “Not reported” indicates that the value was not explicitly listed in the paper.

## 4. The Comprehensive Influences of Fermented Feed for Aquaculture Animals

### 4.1. Feed Efficiency

Numerous studies have demonstrated that fermented feed enhances the feed efficiency (FE) of terrestrial monogastric animals, including pigs [77,78,79], chickens [80], and dogs [81], by improving feed conversion efficiency and promoting weight gain. Similarly, fermented feed enhances FE in aquaculture animals [69]. A summary of the specific influences of fermented feed on aquaculture animal feed efficiency is presented in Table 2. The core mechanism by which fermented feed improves feed efficiency lies in its “pre-digestion” effect and “exogenous enzyme supplementation.” Microorganisms secrete various enzymes (such as proteases, amylases, and cellulases) to break down large molecular substrates into smaller, more readily absorbable nutrients (e.g., small peptides, oligosaccharides, and organic acids). This process directly reduces the animal’s digestive energy expenditure and enhances the apparent digestibility of nutrients [82,83]. However, some research indicates that in Japanese seabass culture, replacing fishmeal with fermented soybean meal resulted in a feed conversion ratio (FCR) significantly higher than that of the fishmeal group but significantly lower than that of the unfermented soybean meal group [72]. This suggests that, for certain aquaculture species, fishmeal remains superior in supporting FE compared to fermented soybean meal, although fermented soybean meal still outperforms its unfermented counterpart. Given the high cost of fishmeal, economically viable alternatives such as fermented feed are critically needed. For example, fermented sesame meal has been shown to significantly increase the profitability of tilapia farming while reducing aquaculture production costs [70]. The addition of fermented sesame meal at 164.3 g/kg significantly improved the economic conversion ratio from $1.37/kg to $1.19/kg in this study.

As shown in Table 2, this effect varied significantly across studies and species. For instance, complete replacement of fishmeal with lactic acid bacteria-fermented silkworm pupae improved the feed conversion ratio in cyprinid fish from 3.16 to 2.10. This improvement is largely attributed to the effective degradation of complex components such as chitin in insect protein and the production of appetite-stimulating organic acids through fermentation [64]. In comparison, the substitution of 40% fishmeal with fermented soybean meal led to a modest elevation in the feed conversion ratio, from 0.80 to 0.95, in pearl gentian grouper [84]. This limited effect may be attributed to two factors: (1) The enzyme profile of the *Bacillus subtilis* strain used in the study may have inadequately degraded anti-nutritional factors in soybean meal, such as trypsin inhibitors; (2) the juvenile gibel carps are more sensitive to amino acid imbalances in plant protein sources, which fermentation may not fully correct. Notably, feed fermented with *Bacillus subtilis* possessing a complex enzyme system has been shown to significantly improve feed conversion efficiency in herbivorous carp [85]; this finding serves as direct evidence for the significance of the “exogenous enzyme supplementation” mechanism.

Fermentation improves the physical and chemical properties of feed by pre-treating it and degrading anti-nutritional factors. Additionally, fermentation produces compounds that enhance feed palatability and improve nutrient utilization [86]. Studies have shown that microbial fermentation of protein feeds generates flavor substances with broad sensory acceptance [87], which stimulate feed intake in aquaculture animals. Furthermore, microbial strains in fermented feed produce enzymes such as hemicellulase, hydrolase, pectinase, lipase, and tannase. Since many aquaculture animals have limited endogenous enzyme production and rely on exogenous enzymes, fermented feed effectively compensates for this deficiency, thereby supporting FCR [85]. Fermentation also disrupts plant cell walls and releases phenolic compounds, enhancing nutrient bioavailability and antioxidant capacity [88], ultimately improving FCR.

The degree of feed efficiency improvement is contingent on an interplay of microbial function, host physiology, and process control. Notably, enzymatic strains outperform mere acidifiers in directly boosting FCR, particularly for complex substrates [89]. Furthermore, the digestive adaptation of the target species determines the value added by fermentation, with carnivores gaining less than species dependent on pre-processed feed. Crucially, process parameters exhibit a dose-effect [90]; deviation from the optimal range—whether leading to residual anti-nutrients or nutrient loss—compromises FCR outcomes. This underscores that maximizing benefit requires identifying the precise fermentation window tailored to the specific substrate–microbe combination [91].

Currently, research on the influences of fermented feed on aquaculture animals predominantly focuses on fish, with relatively few studies addressing crustaceans such as shrimp and crabs, or shellfish. Notably, the optimal substitution levels of fermented protein feed vary across species.

**Table 2:** Influences of fermented feed on the feed efficiency of aquaculture animals.

Fermentation Substrate	Experimental Animals	Proportion	Results	References
Soybean meal	Largemouth bass (*Micropterus salmoides*)	Replace 50% of Fishmeal	FCR from 1.2 to 0.8 *	[92]
Soybean meal	Juvenile coho salmon (*Oncorhynchus kisutch*)	Add 10%	FCR from 1.64 to 1.53 *	[93]
Soybean meal	Juvenile barramundi (*Lates calcarifer*)	Replace 75% of Fishmeal	FCR from 1.47 to 1.46	[94]
Poultry by-product meal	Nile tilapia (*Oreochromis niloticus*)	Add 10%	FCR from 1.37 to 1.24 *	[67]
Silkworm pupae	30% each of catla (*C. catla*), mrigal (*C.* *mrigala*) and rohu (*L. rohita*) with 10% silver carps (*H. molitrix*)	Replace 100% of Fishmeal	FCR from 3.16 to 2.10 *	[64]
Lemon peel	Orange-spotted grouper (*Epinephelus coioides*)	Add 5%	FCR from 1.03 to 0.86 *	[95]
Soybean meal	Pacific white shrimp (*Litopenaeus vannamei*)	Replace 60% of soybean meal	FCR from 1.40 to 1.20 *	[66]
Astragalus membranaceus	Juvenile tiger grouper (*Epinephelus fuscoguttatus*)	Add 4%	FCR from 1.31 to 1.24	[96]
Corn cob	Nile tilapia (*Oreochromis niloticus*)	Add 2%	FCR from 1.05 to 0.85 *	[68]
Palm Kernel Meal	Sex-reversed red tilapia (*Oreochromis niloticus* × *O. mossambicus*)	Replace 25% of Fishmeal and soybean meal	FCR from 1.70 to 1.64	[97]
soybean meal	Pompano (*Trachinotus blochii*)	Replace 50% of the fish meal	FCR from 2.35 to 2.19 *	[98]
Water Spinach Meal	Female stinging catfish (*Heteropneustes fossilis*)	Replace 100% of fishmeal	FCR from 2.20 to 1.69 *	[99]
Soybean Meal	African catfish (*Clarias gariepinus*)	Replace 70% of Fishmeal	FCR from 1.36 to 1.27 *	[100]
Soybean Meal	Pearl gentian grouper (*Epinephelus fuscoguttatus* × *E.lanceolatus*)	Replace 40% of fishmeal	FCR from 0.80 to 0.95 *	[77]
15% fish meal, 40% kelp powder, 40% soybean meal, 5% spirulina	Abalone(*Haliotis discus hannai*)	Replace all the protein feed	FCR from 1.58 to 1.20 *	[75]
Pomelo Peel and Soybean Meal	Large yellow croaker (*Larimichthys crocea*)	Replace 100% of the soybean meal	FCR from 1.14 to 1.09	[74]

Note: Lower FCR values indicate better performance. * Significant difference (*p* < 0.05).

### 4.2. Immune Function

Recent studies have demonstrated that fermented feed enhances the immune capacity of aquaculture animals [72]. Fermented feed enhances immune function via multiple mechanisms. These include supplying low-molecular-weight immune-active compounds such as peptides and polysaccharides, introducing probiotic strains that colonize the intestine and stimulate gut immune tissues, and indirectly relieving immunosuppression through improved antioxidant capacity [101]. Fermentation serves as a pretreatment process that degrades macromolecular compounds into smaller molecular components. High-molecular-weight compounds typically exhibit poor water solubility, complex structures, and conformations, limiting their ability to traverse tissue barriers, enter cells, or bind receptors to elicit biological functions [96]. In contrast, low-molecular-weight compounds possess greater water solubility and lower viscosity, facilitating easier absorption and higher bioavailability in vivo. Moreover, these smaller molecules display stronger affinity for phagocytes, thereby enhancing immune activation [102].

Probiotics used in fermentation can enhance the immune function of aquaculture animals by inhibiting pathogen colonization through multiple mechanisms, including promoting antibody production, competing for adhesion sites and nutrients, and exerting bactericidal effects [103]. Additionally, an animal’s antioxidant capacity is closely linked to its immune function, as prolonged oxidative stress can disrupt the immune system and impair immune defense [104]. Several studies have demonstrated that fermented feed improves the antioxidant capacity of aquaculture animals [93,105], which likely contributes to the observed enhancement of immune function. A summary of the influences of fermented feed on the immune responses of aquaculture animals is presented in Table 3.

Results from certain studies presented in Table 3 indicate that IgM levels increased threefold [105]. This effect may be attributed to a potent activation of humoral immunity, likely associated with specific immunostimulatory polysaccharides generated during fermentation or a high dose of live bacteria. This is supported by research showing an 11% increase in lysozyme activity [66], This may result from small-molecule nutrients improving overall health status or from probiotics mildly stimulating innate immunity. Concurrently, some studies have indicated a reduction in inflammatory cytokine mRNA levels [75]. This clearly indicates that fermented feed (via probiotics or anti-inflammatory metabolites) exerts an inhibitory effect on intestinal or systemic inflammation. The response of immune markers varies considerably across studies, largely because different indicators exhibit distinct sensitivities and magnitudes of response to stimulation. Short-term trials may only detect changes in innate immune parameters (e.g., lysozyme activity), whereas a significant increase in antibody levels typically requires a longer experimental duration.

**Table 3:** Influences of fermented feed on the immune function of aquaculture animals.

Fermentation Substrate	Experimental Animals	Proportion	Results	References
Moringa oleifera	Nile tilapia (*Oreochromus niloticus*)	Add 10%	IgM increased significantly by 300%	[105]
Soybean meal	Juvenile coho salmon (*Oncorhynchus kisutch*)	Add 10%	SOD, CAT, GSH-PX, GST, and Nrf2 were higher	[93]
black soldier fly larvae	Asian swamp eel (*Monopterus albus*)	Replace 24.1% of fishmeal	Nuclear factor-kappa B was increased by 250%	[65]
Lemon peel	Orange-spotted grouper (*Epinephelus coioides*)	Add 3%	The hepatic malondialdehyde decreased by 29.7%	[95]
Soybean meal	Pacific white shrimp (*Litopenaeus vannamei*)	Replace 60% of soybean meal	Lysozyme increased by 11%	[66]
Poultry by-product meal	Nile tilapia (*Oreochromis niloticus*)	Add 10%	Phagocytic increased by 16%	[67]
Corn cob	Nile tilapia (*Oreochromis niloticus*)	Add 2%	Lysozyme increased by 34%	[68]
Soybean Meal	Pearl gentian grouper (*Epinephelus fuscoguttatus* × *E.lanceolatus*)	Replace 40% of fishmeal	IL-17 increased by 35%	[77]
15% fish meal, 40% kelp powder, 40% soybean meal, 5% spirulina	Abalone (*Haliotis discus hannai*)	Replace all the protein feed	mRNA of NF-κB and IL-16 reduced by 75% and 63%	[75]
Pomelo Peel and Soybean Meal	Large yellow croaker (*Larimichthys crocea*)	Replace all soybean meal	mRNA of ZO -1 increased by 55%	[74]
Moringa (*Moringa* *oleifera Lam*.) leaves	Juvenile gibel carp (*Carassius auratus gibelio var.* CAS III)	Replace 60% of the fish meal.	IgM increased by 7%	[85]

### 4.3. Gut Health

The intestine, as the largest immune organ in animals, plays a critical role in digestion, nutrient absorption, and immune function [106], which in turn influence feed efficiency and product quality [107]. The maintenance of intestinal health by fermented feed is centered on “microbial-host interaction.” This is primarily mediated through: (1) probiotic-driven competitive exclusion and colonization resistance; (2) fermentation-derived metabolites that serve as an energy source for enterocytes and enhance mucosal barrier integrity; and (3) lowering of the gut lumen pH to suppress pathogenic bacteria [108]. In mammals, the intestine also affects emotional states via the “Microbiota–Gut–Brain Axis” [109] and is involved in the regulation of various diseases [110,111,112,113]. Moreover, it exerts long-term effects on the growth and development of offspring stem cells [114] and neural development [115]. In livestock such as sheep, maternal gut microbiota can be transmitted to offspring through the uterus, with microbial colonization of the fetal intestine beginning in utero [116]. Similarly, sows’ consumption of fermented feed has been shown to protect offspring from intestinal inflammation by modulating the gut microbiota [108]. However, no evidence currently supports maternal gut microbiota transmission to offspring in aquaculture species, possibly due to their non-mammalian physiology and comparatively shorter intestines. Regardless of species, probiotics are widely reported to regulate intestinal health [117,118,119]. Serving as carriers in the fermentation process, probiotics enable fermented feed to significantly contribute to intestinal health in animals. Numerous studies have documented the beneficial influences of fermented feed on the intestinal health of aquaculture animals [11,17,107]. A summary of these influences is presented in Table 4.

Evaluating the impact of fermented feed on gut health requires an integrated assessment across two complementary dimensions: morphological structure and microecological balance [120]. These are not isolated but are intricately linked through functional interplay and causal relationships. On one hand, an intact intestinal structure with well-developed villi provides a larger colonization surface area and a stable anaerobic environment for beneficial microorganisms. On the other hand, a healthy gut microbiota—particularly lactic acid bacteria and others—produces short-chain fatty acids (SCFAs, such as butyrate), which directly energize intestinal epithelial cells and serve as key signaling molecules for their proliferation and the maintenance of barrier integrity [37]. Some studies presented in Table 4 demonstrates a notable increase in villous height across certain species [76]. This is attributed to the role of SCFAs—particularly butyrate—in stimulating the proliferation of intestinal epithelial cells. Consistent with this, some studies have reported a substantial increase in the abundance of *Lactobacillus* and *Lactococcus* [121]. The results demonstrate successful modulation of the intestinal microbiota structure by probiotics from the fermented feed. In line with this shift, a corresponding decrease in certain pathogenic genera was reported in some studies [66]. This reflects the competitive exclusion by probiotics or the direct antibacterial effect of fermentation metabolites. Notably, an anomalous finding from one study reported a 34% reduction in villus height [65]. The observed negative effect could stem from gut-irritants generated by unsuitable materials or processes, or from an excessive substitution rate causing nutrient imbalance in the Asian swamp eel (*Monopterus albus*)—a result that paradoxically highlights the necessity of tailored optimization. Collectively, the data show that fermented feed can affect gut structure and microbiota in synergistic, independent, or even contradictory ways. The final impact hinges on establishing a positive feedback loop where fermentation products simultaneously support intestinal tissue and foster a beneficial microbial community. Moving forward, studies should simultaneously track both dimensions and utilize tools like correlation analyses and germ-free models to clarify causality and pave the way for targeted gut health management.

**Table 4:** Influences of fermented feed on the gut health of aquaculture animals.

Fermentation Substrate	Experimental Animals	Proportion	Results	References
Black soldier fly larvae	Asian swamp eel (*Monopterus albus*)	Replace 24.1% of fishmeal	The height of villi reduced by 34%	[65]
Soybean meal	Pacific white shrimp (*Litopenaeus vannamei*)	Replace 60% of soybean meal	Acinetobacter significantly reduced	[66]
Astragalus membranaceus	Juvenile tiger grouper (*Epinephelus fuscoguttatus*)	Add 4%	The diversity and richness increased	[97]
Soybean meal	Turbot (*Scophthalmus maximus* L.)	Replace 45% of fishmeal	The heights of villi and microvilli increased by 23% and 37%	[76]
Soybean meal	Atlantic salmon (*Salmo salar*)	Replace 30% of fish meal	Lactobacillus and Lactococcus increased by 120% and 100%	[121]
Water spinach meal	Female stinging catfish (*Heteropneustes fossilis*)	Replace 50% of fishmeal	The villus structure was improved	[100]
Soybean meal	African catfish (*Clariasm gariepinus*)	Replace 70% of fishmeal	Staphylococcus succinus increased by 75%	[101]
Soybean meal	Pearl gentian grouper (*Epinephelus fuscoguttatus* × *E.lanceolatus*)	Replace 20% and 40% of fishmeal	α-diversity index both increased	[84]
15% fish meal, 40% kelp powder, 40% soybean meal, 5% spirulina	Abalone (*Haliotis discus hannai*)	Replace all the protein feed	Shannon index increased by 4%	[75]
Pomelo peel and soybean meal	Large yellow croaker (*Larimichthys crocea*)	Replace all the soybean meal	Beneficial bacteria increased	[74]

### 4.4. Quality of Aquaculture Animal Products

In the contemporary Chinese aquaculture product market, consumer preferences have shifted from merely “eating enough” to “eating well,” placing greater emphasis on the quality of aquaculture animal products. Current research on the application of fermented feed in aquaculture animals primarily addresses feed efficiency, immune function, and intestinal health, with relatively few studies examining product quality. This contrasts with research in terrestrial livestock, where numerous studies have documented the positive influences of fermented feed on product quality in animals such as pigs and poultry [39,122,123,124]. As animal feed directly influences meat quality [125], fermentation—a feed processing method that enhances nutrient bioavailability [126]—can improve meat flavor and yield greater economic benefits. Evaluating the impact of fermented feed on the product quality of aquatic animals requires a comprehensive assessment across three dimensions: consumer perception, nutritional value, and processing/storage characteristics. Flavor (particularly umami) and texture constitute the primary sensory attributes determining consumer acceptance. Nutritional components (e.g., amino acid and fatty acid profiles) and food safety aspects (such as oxidative stability) are core nutritional and safety attributes, while processing yield influences economic viability. Different indicators carry varying weights in determining final quality. For instance, a marginal increase (e.g., 3–5%) in glutamate—a key umami substance—may improve flavor far more significantly than a substantial rise in certain non-flavor amino acids. The mechanism by which fermented feed enhances product quality primarily involves the targeted regulation of animal metabolism and the deposition of flavor precursors [102]. Amino acid composition is a crucial parameter for assessing meat nutrition and quality, as amino acids in muscle tissue significantly affect product characteristics, particularly flavor-related compounds such as glutamic acid and aspartic acid [127]. Notably, glutamic acid, known as the “source of umami,” also serves as a key precursor for the synthesis of other amino acids [128], warranting focused evaluation. However, existing studies on fermented feed’s impact on aquaculture animal product quality have largely concentrated on amino acid deposition. Future research should adopt a more comprehensive approach to evaluating product quality. The influences of fermented feed on the product quality of aquaculture animals are summarized in Table 5.

Changes in amino acid content in some studies, such as increased Ser, Asp, and Glu [107], suggest that fermented feed either optimizes the animal’s overall amino acid metabolism or enhances the bioavailability of these flavor-associated amino acids from the feed itself. Research showing a 50% reduction in lipid peroxides [10] clearly indicates that fermented feed enhances the animal’s antioxidant capacity, thereby reducing oxidative rancidity of muscle fat, which benefits shelf life and flavor stability. Another study reported no change in the muscle fiber of Asian swamp eel (*Monopterus albus*) [65], indicating that the fermented feed primarily improves biochemical composition rather than muscle structure. Future research should move beyond single amino acid metrics to systematically analyze more comprehensive quality dimensions—such as volatile flavor compounds, fatty acid profiles, and muscle texture—and explore their links with specific fermentation metabolites.

**Table 5:** Influences of fermented feed on the quality of aquaculture animal products.

Fermentation Substrate	Experimental Animals	Proportion	Results	Degree of Importance	References
Black soldier fly larvae	Asian swamp eel (*Monopterus albus*)	Replace 24.1% of fishmeal	No change in muscle fiber size	Low	[65]
Complete feed	Chinese mitten crab (*Eriocheir sinensis*)	Ferment the whole feed	Ser and Asp increased by 130% and 30%	Moderate	[107]
Compost	Carp(*Cyprinus carpio*)	Add 20%	Lipid peroxides reduced by 50%	High	[10]
sugar kelp (*Saccharina la latissima*)	Atlantic salmon (*Salmo salar* L.)	Add 4%	Iodine increased by 26%	High	[129]
Palm kernel meal	Sex-reversed red tilapia (*Oreochromis niloticus* × *O. mossambicus*)	Replace 50% of fishmeal and soybean meal	Protein synthesis capacity increased by 20%	Moderate	[98]
15% fish meal, 40% kelp powder, 40% soybean meal, 5% spirulina	Abalone (*Haliotis discus hannai*)	Replace all the protein feed	Glutamic acid increased by 3%	Very high	[75]
Animal protein wastes	Carnivorous catfish (*Mystus vittatus*)	Replace 100% of fishmeal	Arg increased by 8%.	Moderate	[130]

## 5. Mechanisms of Action of Fermented Feed

The multiple benefits of fermented feed for aquatic animals arise from the interplay of microbial metabolism, substrate transformation, and host physiological responses, rather than from any single process. Understanding its mechanism of action necessitates an integrated approach that begins with the fermentation process, traces subsequent changes in product composition, and ultimately links these changes to physiological and metabolic outcomes at the animal level.

### 5.1. Functional Differentiation and Product Orientation of Microbial Strains

The choice of microbial strain is the core driver of fermentation, defining the metabolic routes and shaping the final product profile, which ultimately results in different physiological outcomes: (1) Fermentation driven by lactic acid bacteria: The mechanism centers on the rapid generation of acids (primarily lactic and acetic acid). This rapidly lowers the environmental pH, thus inhibiting the growth of pathogens like *E. coli* and *Salmonella* [131]. This process not only enhances feed preservability by preventing secondary fermentation but also produces organic acids that serve as direct energy substrates for intestinal epithelial cells, thereby further strengthening the intestinal barrier function [132]. This accounts for the observation in Table 3 (Immune Function) that LAB-fermented feed is typically accompanied by elevated lysozyme activity and reduced expression of inflammatory factors, such as NF-κB. (2) Bacillus-dominated fermentation: This process involves the secretion of abundant extracellular enzymes (e.g., proteases, amylases, cellulases), which break down macromolecules such as proteins, starch, and fiber into small peptides, oligosaccharides, and soluble fiber. This “pre-digestion” significantly improves substrate digestibility, making it particularly suitable for juveniles or certain species (e.g., shrimp) with insufficient endogenous enzyme secretion [133]. Therefore, the marked improvement in FCR shown in Table 2—for instance, the FCR of fermented silkworm pupa decreased from 3.16 to 2.10—is strongly associated with the application of Bacillus or compound microbial agents containing *Bacillus subtilis*. (3) Yeast-dominated fermentation: This process generates ethanol and CO_2_ while also synthesizing various B vitamins, glutathione, and flavor compounds (e.g., esters and alcohols). Additionally, yeast cell wall components (β-glucans, mannan oligosaccharides) are recognized immunostimulants that can activate macrophages and enhance antibody responses [32]. This mechanism likely explains the elevated immunoglobulin (IgM) levels observed in yeast-fermented groups (e.g., fermented poultry by-products) in Table 3.

### 5.2. Bioactive Pathways of Critical Components

Specific components in the fermentation end-products act on the host through the following pathways: (1) Small-molecule nutrients and bioactive peptides: Enzymatic hydrolysis of proteins releases a large number of small peptides and free amino acids, which are absorbed at a higher rate than intact proteins [134]. Additionally, this process may yield bioactive peptides with antimicrobial and immunomodulatory functions, such as antibacterial peptides derived from fermented soybean meal. These effects are directly linked to the improved muscle amino acid profile (e.g., serine, aspartic acid) and enhanced product flavor documented in Table 5. (2) Organic acids and phenolic compounds: This pathway involves two main actions: organic acids (e.g., lactic, acetic) not only reduce pH but also regulate gut microbiota to favor beneficial bacteria (e.g., Lactobacillus). Meanwhile, phenolic substances (e.g., from fermented lemon peel or moringa) released during fermentation possess antioxidant activity, mitigating oxidative stress [135]. This mechanism corresponds to the increased activity of antioxidant enzymes (SOD, CAT) reported in Table 3, which may explain the observed improvement in antioxidant status. (3) Exogenous enzymes and probiotics: Fermented feed inherently serves as a carrier for both enzyme preparations and probiotics. The exogenous enzymes directly assist in digestion, while viable probiotics can colonize or transiently reside in the intestinal tract [136]. Through mechanisms such as competitive exclusion and bacteriocin production, they inhibit pathogenic bacteria and modulate the host’s immune response.

### 5.3. Preliminary Analysis of Species-Specific Response Mechanisms

The differential responses of various aquatic animals to the same fermented feed stem from their distinct physiological and digestive traits. (1) Fish vs. Crustaceans: Fish possess strong gastric acid secretion and relatively long intestines, enabling high tolerance and efficient utilization of organic acids and pre-digested proteins [137]. In contrast, crustaceans (e.g., shrimp) have a simpler gastric gland structure and short intestinal tracts, making them more reliant on exogenous enzymes for feed pre-decomposition [138]. Consequently, Bacillus-fermented feed rich in proteases and amylases likely produces more pronounced improvements in FCR for shrimp (e.g., Pacific white shrimp FCR decreased from 1.40 to 1.20 in Table 2), whereas the effect may be more moderate in certain fish species. (2) Omnivorous vs. Carnivorous fish: Omnivorous fish (e.g., tilapia), with their more complex gut microbiota, are likely more efficient at utilizing short-chain fatty acids and microbial protein produced during fermentation [139]. Conversely, carnivorous fish (e.g., sea bass) have exceptionally high requirements for protein quality and amino acid balance [140]. Should fermentation lead to the degradation of certain limiting amino acids (e.g., lysine), it may result in poor replacement efficacy or even a slight increase in FCR, as observed in some cases in Table 2.

The efficacy of fermented feed results from the synergistic interplay between its “components” (bioactive products) and “carriers” (live microbes), which is further modulated by the physiological status of the host. Future research must move beyond a “black box” perspective and adopt an integrated approach that combines targeted fermentation—tailoring processes to meet species-specific needs—with mechanistic validation, which involves quantifying key products and tracing them in vivo metabolic fate. This shift is essential to advance from empirical application to precise nutrition.

## 6. Critical Assessment of the Current State of Fermented Feed Research

Although this review incorporates a substantial body of recent research on the application of fermented feed in aquatic animals, it must be noted that the available evidence exhibits significant heterogeneity and limitations in terms of methodology, experimental design, and interpretation of results. The following systematic assessment from three perspectives—research quality, variability in outcomes, and research gaps/cognitive limitations—aims to clarify the boundaries of current knowledge and identify priority directions for future studies.

### 6.1. Heterogeneity in Study Design and Methodological Quality

Most studies in this field are feeding trials focused on measuring phenotypic parameters such as growth performance and immune indices. However, they are typically constrained by the following methodological limitations: (1) Small sample sizes, with some studies using fewer than 30 individuals per group, thereby compromising statistical power [67]. (2) Incomplete documentation of fermentation processes is a common issue. As shown in Table 1, key parameters (e.g., moisture content [74], viable cell titer [71,84]) are frequently unreported in some studies, which significantly undermines the reproducibility of the experiments. (3) Inadequate control groups. A few studies established only a blank control, lacking comparisons with an unfermented raw material control [68] or different fermentation strains [93].

### 6.2. Heterogeneity of Study Results

Significant variations exist across studies, particularly for metrics such as FCR and immune parameters. For instance, the use of fermented soybean meal in pomfret improved the FCR from 2.35 to 2.19 [99], while in juveniles of barramundi (Lates calcarifer), it only showed a marginal reduction from 1.47 to 1.46 [94]. This variability may originate from the following factors: (1) Species specificity: Aquatic animals differ greatly in digestive physiology, enzyme systems, and gut microbiota composition, leading to divergent responses to the same fermented substrate. (2) Inconsistent fermentation conditions: As indicated in Table 1, variations in temperature (25–40 °C), duration (2–10 days), and microbial consortia can result in distinct metabolite profiles, thereby altering the nutritional properties of the product. (3) Substitution ratio and basal diet formulation: Uncontrolled variables such as fishmeal replacement level, dietary amino acid balance, and energy density may either mask or amplify the true effects of fermented feed.

### 6.3. Knowledge Gaps and Current Limitations

Current research exhibits two prominent biases: species imbalance and superficial mechanistic investigation. (1) Uneven species coverage: As shown in Table 2, over 60% of data originate from fish (with tilapia being the most studied), while research on crustaceans (shrimp, crabs) and mollusks is severely limited. This imbalance constrains the generalizability of conclusions across the broader aquaculture industry. (2) Lack of mechanistic depth: The vast majority of studies have remained largely descriptive, lacking quantitative analysis of fermentation products (e.g., specific organic acids, bioactive peptides, enzymes) and in-depth exploration of their interactions with the host gut microbiota. For instance, reports on gut health improvement often rely on morphological parameters (villus height) or microbiome diversity indices, with few studies tracking the dynamics and signaling pathways of specific metabolites like short-chain fatty acids. (3) Gap in long-term and safety data: Most trials last only 4–12 weeks, failing to assess cumulative effects over entire production cycles or multiple generations. Furthermore, research evaluating the risks of fermentation failure (e.g., contamination, mycotoxin accumulation) or nutrient loss due to over-fermentation is virtually absent.

In summary, the field remains in a transitional stage from descriptive observation to mechanistic understanding. Future research must focus on four key directions: standardizing fermentation processes, strengthening cross-species comparisons, integrating multi-omics technologies, and conducting long-term safety assessments, thereby establishing a predictable and regulatable application system for fermented feed.

## 7. Safety and Risk Considerations of Fermented Feed

Despite the considerable potential of fermented feed to enhance nutrition and health, its production and application are not without risks. Systematic identification and management of these risks are prerequisites for the sustainable adoption of this technology. This section will analyze the issue from three critical dimensions: microbial safety, process controllability, and application efficacy.

### 7.1. Microbial Safety: Contamination and Toxin Risks

Fermentation is inherently an ecosystem succession driven by microorganisms, involving competition between target strains (probiotics) and contaminants (including potential pathogens). The primary safety risks include: (1) Pathogenic Microbial Contamination: Inadequate hygiene control during production can lead to contamination by pathogens such as *Salmonella* and *E. coli*. More notably, certain fermentation conditions (e.g., high temperature, high moisture) may favor the proliferation of pathogenic strains within the Bacillus genus or clostridia (e.g., Clostridium perfringens). (2) Accumulation of Mycotoxins and Biogenic Amines: These represent two critical chemical hazards. If raw materials are already contaminated with mold (e.g., aflatoxins) or become contaminated with toxin-producing fungi (e.g., certain Aspergillus, Penicillium spp.) during fermentation, toxins may accumulate or be transformed in the feed. Concurrently, during the fermentation of protein-rich materials, decarboxylase activity from some microbes can convert free amino acids into biogenic amines like histamine and tyramine. Excessive intake can cause toxic reactions in animals. (3) Quality Control Measures: Ensuring safety requires implementing a comprehensive “farm-to-feed” control strategy: ① Raw Material Screening and Pretreatment: Strictly test and control the baseline levels of mycotoxins in ingredients. ② Process Standardization and Monitoring: Use purified, high-activity commercial starter cultures and strictly control key fermentation parameters (especially temperature and pH) to create an environment unfavorable for contaminants (e.g., rapid acid production by lactic acid bacteria). ③ Final Product Testing: Establish routine testing standards for key pathogens, mycotoxins, and biogenic amines.

### 7.2. Process Risks: Over-Fermentation and Quality Variation

Fermentation is a dynamic bioprocess where both under- and over-fermentation can lead to quality deterioration: (1) Nutrient Loss from Over-fermentation: Excessively long duration or high temperature may, after depleting readily degradable substrates, cause microbes to break down beneficial products already formed. For instance, small peptides and amino acids can be further degraded into ammonia or amines, resulting in nutrient loss and off-odors. Excessive proteolysis, for example, reduces the biological value of the protein. (2) Batch-to-Batch Product Variability: As shown in Table 1, fermentation conditions vary substantially across studies. This wide fluctuation in process parameters directly leads to inconsistency and poor predictability in the final product’s nutritional composition, active substance content, and microbial profile, severely hindering commercial scale-up and standardized application. (3) Defining the Processing Window: The key to mitigating these risks lies in establishing a clear “optimal fermentation window” for each raw material–microbial strain combination. This requires systematic research to define the upper and lower limits for critical parameters like time and temperature, using key nutritional (e.g., acid-soluble protein content), microbiological (e.g., viable cell count), and sensory indicators as endpoints.

### 7.3. Application Risks: Uncertain Efficacy and Ineffectiveness Cases

Fermented feed does not guarantee the promised benefits in all cases, as evidenced by the suboptimal or negative outcomes (e.g., lack of FCR improvement) documented in Table 2. This highlights the application risks: (1) Analysis of Ineffectiveness or Negative Effects: Poor outcomes may stem from: ① Mismatch between microbial strains and raw materials: The selected strains lack the enzymatic repertoire to degrade key antinutritional factors in specific ingredients. ② Exceeding the substitution threshold: Even after fermentation, the amino acid imbalance of certain plant proteins cannot meet animal requirements when included at high inclusion rates. ③ Suboptimal fermentation process: Extreme conditions, as seen in some cases in Table 1, may yield inferior products. ④ Interspecies differences: Physiological limits exist, such as in carnivorous fish, which may have a low tolerance for fermented plant proteins. (2) Prediction and Mitigation Strategies: To mitigate these risks, future R&D should shift toward “precision fermentation.” Prior to large-scale application, targeted in vitro digestion simulations, cell models, or short-term feeding trials should be used to rapidly screen effective strains and optimize processes for specific target species and low-cost ingredients. This predictive approach is preferable to conducting lengthy, blind full-cycle feeding trials.

Acknowledging and systematically managing the safety and application risks of fermented feed is essential for its transition from a “promising technology” to a “reliable industry.” This necessitates multidisciplinary collaboration across microbiology, fermentation engineering, animal nutrition, and feed safety to establish a comprehensive quality control system covering strain safety, process standardization, product specifications, and efficacy verification.

## 8. Challenges and Trends

### 8.1. Existing Challenges and Limitations

Despite the numerous advantages of fermented feed, several challenges remain:

The potential of fermented feed to improve feed efficiency and animal health is well-documented technically. However, its path to large-scale industrial adoption is blocked by two formidable barriers: proving its economic worth and navigating complex regulatory regimes. Research addressing these critical, real-world challenges remains strikingly scarce.

The Missing Economic Case: A major blind spot in the current literature is the lack of rigorous cost–benefit analysis. For adoption, the full financial picture must be clear: the added costs (for starters, equipment, energy, time, QC) must be weighed against tangible benefits. These benefits extend beyond better FCR and health to include potential market premiums for quality. A key question is whether savings from drastically cutting fishmeal use (e.g., halving inclusion rates) and gains from improved performance can offset higher production costs. Without such holistic, cycle-wide economic models, investment and adoption decisions lack a solid foundation.

The Global Regulatory Maze: The international regulatory landscape for fermented feed—a product containing live microbes—is complex and fragmented. Strain approval is tightly controlled: the EU, US, and China each have distinct systems governed by different agencies (e.g., EFSA/GMO panels, FDA/EPA, MOA) and legal frameworks. This heterogeneity means that a product legally marketed in one region may face a lengthy, expensive re-approval process in another, creating a significant non-technical barrier to global trade and widespread application.

Significant challenges remain in both process control and mechanistic understanding. The fermentation process is susceptible to contamination by spoilage microorganisms, which can lead to feed deterioration and even toxin (e.g., mycotoxins, biogenic amines) production, resulting in economic losses. Currently, as indicated in Table 1, the vast majority of studies employ vastly different microbial strains, inoculation levels, and fermentation conditions, reflecting a critical lack of standardized protocols. A potential solution is to establish a whole-process quality control system encompassing strain selection, fermentation management, and final product specifications. Preliminary progress is noted, as a few studies have begun utilizing commercial, patented strains; however, the industry as a whole still lacks universally accepted safety and production standards.

Despite numerous observed phenotypic benefits, identifying the specific active components (e.g., particular peptides, organic acids) within fermented feed and elucidating their signaling pathways affecting host physiology remain largely a “black box.” This knowledge gap leads to a degree of blindness in formula design, hindering the achievement of precision fermentation tailored for specific farming objectives, such as disease resistance or meat quality improvement. A key research limitation is that current studies are predominantly led by animal nutritionists, lacking deep interdisciplinary integration with fields like microbiomics, metabolomics, and molecular biology. Methodologically, research still relies heavily on feeding trials and phenotypic measurements.

Processes proven successful at the laboratory scale often encounter significant engineering challenges when scaled up to industrial production, including uneven mass/heat transfer, heightened contamination risks, and increased energy consumption. Moreover, there is a lack of systematic case studies and validated economic models to determine whether the added costs in time, equipment, and energy from fermentation are offset by gains in feed efficiency, fishmeal replacement, and potential product premiums. This uncertainty constitutes a major barrier to corporate investment.

While the use of compound microbial consortia is often advocated, the interactions between strains (e.g., symbiosis, competition, antagonism) are poorly understood, making it difficult to identify the dominant functional strains and determine their optimal ratios. Furthermore, although post-fermentation pelleting aids in storage and feeding, the high temperatures involved may inactivate beneficial probiotics and enzymes. Preserving these active components during processing thus remains a critical technical hurdle.

### 8.2. Future Perspectives and Research Directions

Future development trends for fermented feed include the following:

Short-term Objectives (1–3 Years): Strengthening Fundamentals and Standardization

Establishing a Public Database: Create a comprehensive database that correlates variations in raw materials, microbial strains, process parameters, and nutritional outcomes to provide foundational data for the industry.

Formulating Process Standards: Develop reproducible, safe, and controlled standardized fermentation protocols for mainstream ingredients like soybean meal and rapeseed meal.

Expanding Research Beyond Finfish: Prioritize application research on fermented feed for key farmed crustaceans (e.g., shrimp, crabs) to address current data gaps.

Mid-term Objectives (3–5 Years): Deepening Mechanisms and Advancing Intelligence

Elucidating Mechanisms of Action: Employ multi-omics technologies (metagenomics, metabolomics, transcriptomics) to trace the in vivo metabolic fate of fermentation products and clarify the molecular pathways through which they improve health and product quality.

Developing Intelligent Fermentation Systems: Integrate sensors and artificial intelligence for real-time monitoring and control of key parameters (temperature, pH, dissolved oxygen) to achieve precision fermentation and consistent product quality.

Exploring Staged and Targeted Fermentation: Innovate new processes like “aerobic-anaerobic” staged fermentation and research strategies to direct the production of specific functional components (e.g., antimicrobial peptides, immunomodulatory polysaccharides) through process control.

Long-term Vision (5+ Years): Achieving Precision and Sustainability

Advancing Precision Fermented Nutrition: Develop tailored fermented feed formulations based on specific cultured species, growth stages, and even health status, transitioning from a “one-size-fits-all” to a “customized” approach.

Integrating Circular Economy Principles: Systematically evaluate the feasibility of using food industry by-products and agricultural waste as fermentation substrates to reduce costs and environmental footprint.

Building a Holistic Assessment Framework: Establish a comprehensive evaluation model that integrates technical efficacy, economic viability, environmental life-cycle assessment, and regulatory compliance to inform industrial development and policy-making. Collectively, these directions will support the sustainable development and broader application of fermented feed in aquaculture.

## 9. Conclusions

Fermented feed enhances the feed efficiency, immune function, intestinal health, and product quality of aquaculture animals through microbial predigestion and probiotic activity. It offers the potential to reduce costs and improve efficiency, effectively addressing challenges such as poor digestive capacity and low disease resistance in aquaculture species. However, its development remains constrained by challenges including inadequate control of contaminant bacteria, gaps in practitioner expertise, and limited understanding of its underlying mechanisms. Moving forward, emphasis should be placed on mixed-strain fermentation, intelligent production systems, research on crustaceans (such as shrimp and crabs) and the synergistic utilization of multiple raw materials. These strategies will advance fermented feed toward greater efficiency, environmental sustainability, and industrial scalability, thereby providing vital support for the sustainable development of aquaculture.

## Data Availability

The original contributions presented in this study are included in the article. Further inquiries can be directed to the corresponding author.
